# Integrated Transcriptome and Metabolome Analysis Elucidates the Regulatory Networks of Salt Stress Response During Cotton Seed Germination

**DOI:** 10.3390/genes17070761

**Published:** 2026-06-30

**Authors:** Yutao Guo, Li Tian, Shaoyu Cheng, Xiang Ren, Xianliang Zhang

**Affiliations:** 1 Western Research Institute, Chinese Academy of Agricultural Sciences (CAAS), Changji 831100, China; 2 National Key Laboratory of Cotton Bio-Breeding and Integrated Utilization, State Key Laboratory of Crop Stress Adaptation and Improvement, Henan Joint International Laboratory for Crop Multi-Omics Research, School of Life Sciences, Henan University, Kaifeng 475004, China; 3 College of Biology and Food Engineering, Anyang Institute of Technology, Anyang 455000, China

**Keywords:** cotton, early stage of seed germination, salt stress, transcriptome, metabolome

## Abstract

Background/Objectives: Soil salinization constitutes a critical threat to global agriculture, with cotton (*Gossypium* spp.) being highly susceptible. This abiotic stress most severely impacts cotton during the early sowing and seedling stages, compromising stand establishment and early growth. Manifestations of this stress include reduced germination rates, uneven emergence, stunted seedlings, and, ultimately, diminished boll set and fiber yield. Methods: To investigate the molecular basis of salt tolerance in cotton seed germination, we performed integrated transcriptomic and metabolomic profiling of *Gossypium hirsutum* cv. ST022-1056m5 under 150 mM NaCl stress at 24 h, 48 h, and 72 h, finding that salt stress significantly inhibited germination. Differentially expressed genes (DEGs) and differentially accumulated metabolites (DAMs) were identified, followed by functional enrichment and Weighted Gene Co-expression Network Analysis (WGCNA) to construct regulatory networks. Results: Transcriptomics revealed stage-specific differentially expressed genes, with predominant downregulation and enrichment in catalytic/transporter activities. Metabolomics showed distinct reprogramming, with 210 shared differentially accumulated metabolites enriched in lipids, organic acids, terpenoids, and phenolic acids. KEGG analysis highlighted time-dependent pathway shifts: sucrose metabolism and MAPK signaling at 24 h, photosynthesis at 48 h, and cuticular lipid biosynthesis at 72 h. Weighted Gene Co-expression Network Analysis (WGCNA) identified stage-associated modules and hub genes (*GH_A02G0892*, *GH_A08G2853*), and multi-omics integration indicated the strongest transcript–metabolite coordination at 24 h. Conclusion: Our study reveals dynamic molecular reprogramming underpinning stage-specific salt adaptation in germinating cotton seeds. These identified DEGs, DAMs, and hub genes represent promising candidate targets for molecular breeding and offer a crucial genetic basis for improving salt tolerance in cotton.

## 1. Introduction

Cotton, as the world’s most important source of natural fiber and a significant oilseed crop, has production stability that is crucial to the national economy and people’s livelihoods [1]. However, the problem of salinization caused by worldwide irrigated agriculture, as well as primary soil salinization in arid and semi-arid regions, is becoming increasingly severe [2]. Salt stress suppresses cotton growth via osmotic, ionic, nutrient, and oxidative stresses (secondary), reducing lint yield, degrading fiber quality, and threatening production security [3,4,5,6,7]. Therefore, analyzing the mechanisms underlying cotton’s response to salt stress and enhancing its salt tolerance have become both a frontier and an urgent demand in agricultural scientific research.

Cotton exhibits stage-specific sensitivity to salt stress, with the seed germination and early seedling stages being the most vulnerable [8,9,10,11,12,13]. At this phase, small plant size, immature protective mechanisms, and fragile autotrophic transition coupled with high metabolic activity make seedlings prone to salt-induced damage, manifested as reduced germination rate, uneven emergence, deformity, or death, irreversibly compromising the final yield [14,15,16]. Thus, elucidating cotton seed responses during germination under salt stress is key to “targeting the source” for breeding salt-tolerant varieties and optimizing stress-resistant cultivation. Existing studies focus on salt stress effects on germination metrics (e.g., rates, seedling growth) [13,17] yet rely mostly on low-temporal-resolution observations (days/weeks) [18,19]. A gap exists in understanding real-time seed internal dynamics during the critical 24–72 h post-salt-stress window—the decisive “make-or-break” phase for germination.

Seed germination is an ordered, energy-intensive physiological process spanning from water imbibition to radicle emergence [20]. In cotton, this process is conventionally divided into three phases: imbibition (0–24 h), activation (24–48 h), and radicle elongation (≥48 h) [21]. Salt stress poses a severe threat during the initial “golden window” where irreversible damage occurs [21]. During imbibition, reduced soil water potential and passive Na^+^/Cl^−^ influx trigger ion toxicity and osmotic stress [22,23]. The subsequent activation phase acts as a “decision-making period”; here, the cumulative effects of ionic toxicity, impaired mitochondrial function, and oxidative stress converge to block radicle emergence [24,25].

However, a major gap remains in understanding how these stressors are systemically coordinated at the molecular level. Conventional studies relying on isolated physiological indicators or low-temporal-resolution sampling fail to capture the multi-layered regulatory networks (perception, signaling, transcription, and metabolism) governing salt tolerance [20,25]. While transcriptomics reveals differential gene expression, sparse time-course data often obscure the distinction between immediate stress responses and secondary adaptations [26].

In this study, we integrated transcriptomic and metabolomic profiling to provide a holistic view of cotton germination under salinity. By focusing on the critical 1–3-day window, this multi-omics approach elucidates the dynamic reprogramming of gene expression and metabolic fluxes. Our findings move beyond mere description to uncover the mechanistic basis of salt tolerance, offering vital targets for breeding salt-tolerant varieties and optimizing cultivation strategies.

## 2. Materials and Methods

### 2.1. Plant Materials and Growth Conditions

To investigate salinity tolerance mechanisms in *G. hirsutum*, salt-tolerant cotton cultivar (*G. hirsutum* cv. ST022-1056m5) seeds were initially subjected to the delinting treatment using concentrated sulfuric acid. Subsequently, plump and uniform seeds were selected and used for a germination experiment, with treatments including deionized water or a 150 mM NaCl solution in the dark at 28 °C for germination. Six independent biological replicates of 100 seeds each were prepared per treatment. Three replicates were germinated in a chamber (28 °C, dark conditions) for three time points (24 h, 48 h, 72 h) to record germination (defined as radicle emergence ≥ 2 mm).

### 2.2. RNA-Seq Analysis

To systematically identify salt stress-responsive genes in the early stage of germination. Total RNA was extracted from the powder for transcriptome analysis, with three biological replicates performed per treatment. The RNA-seq data were generated using the Illumina NextSeq2000 platform with the P4 XLEAP-SBS Reagent Kit (50 cycles) (Illumina, Shanghai, China). The resulting data were subjected to bioinformatics analysis using the Majorbio Cloud Platform (Shanghai, China) and mapped to the annotated *G. hirsutum* TM-1 reference genome (http://cotton.zju.edu.cn/source/TM-1_V2.1.fa.gz, accessed on 16 January 2026) using HISAT2 v2.0.5. Transcriptome sequencing of 18 samples (experiments were performed with *n* = 3 biological replicates per experimental group) was completed, generating a total of 116.03 Gb of clean data, with each sample containing at least 6.1 Gb of clean data and the Q30 base percentage exceeding 96.08% (Table S6). The DESeq package in R was employed to analyze the difference in gene expression between water treatment and 150 mM NaCl solution treatment, and genes with the value of |log_2_ (fold change)| ≥ 1 and *p* value  <  0.05 were defined as differentially expressed genes (DEGs) [27]. Gene Ontology (GO) annotations were assigned using Blast2GO, followed by enrichment analysis with TopGO (R package). The gene list and the number of each term were calculated using the differentially expressed genes annotated by GO terms [28]. Hypergeometric testing (*p* < 0.05) and FDR < 0.05 were the identification criteria. Identified DEGs were annotated using the newly updated KEGG and GO databases and mapped to the KEGG and GO pathways [29].

### 2.3. Widely Targeted Metabolome Analysis

To characterize the metabolic responses in the early stage of germination under salt stress, cotton seed samples after 24 h, 48 h, and 72 h of treatment with 150 mM NaCl solution and water were harvested in three biological replicates and then immediately frozen using liquid nitrogen. This characterization was conducted by Majorbio Bio-Pharm Technology Co., Ltd. (Shanghai, China). Samples were ground into fine powder using liquid nitrogen and then placed in 70% aqueous methanol. After centrifugation at 10,000× *g* for 10 min, all supernatants were combined and filtered through a 0.22 µm pore size membrane and then analyzed using a UPLC-ESI-MS/MS system (UPLC, ExionLC™, Thermo Scientific™ Orbitrap™ Astral™, Shanghai, China) and a tandem mass spectrometry system. The reference standard was used for quality control. Then, the raw data were processed using Progenesis QI (Waters Corporation, Milford, MA, USA) for peak detection, extraction, alignment, and integration. Metabolite annotation was performed by matching against the HMDB (http://www.hmdb.ca/, accessed on 16 January 2026), Metlin (https://metlin.scripps.edu/, accessed on 16 January 2026), and Maji’s database (Shanghai, China). Features with a missing value rate > 20% within any sample group were removed. Missing values in the remaining data were then imputed with the minimum value across all samples. The peak response intensities were normalized using the total sum normalization method. Furthermore, variables with a relative standard deviation (RSD) > 30% in the QC samples were excluded. Based on the identified metabolites, principal component analysis (PCA) was conducted using the FactoMineR and factoextra packages in R to visualize sample clustering patterns [30,31]. Orthogonal Partial Least Squares Discriminant Analysis was performed to determine the DAMs using a threshold of |log_2_FoldChange| > 1 and variable importance in project ≥ 1. Based on the cloud database, identified metabolites were annotated using the newly updated KEGG compound database and mapped to the KEGG pathways.

### 2.4. Integrated Transcriptomic and Metabolomic Analysis

To further explore the relationship between salt-responsive metabolic and transcriptomic changes in the early stage of germination under salt stress, we performed KEGG pathway analysis to identify pathways that were co-enriched by differentially expressed genes (DEGs) and differentially accumulated metabolites (DAMs). Pathway enrichment analysis was conducted, and pathways with a *p*-value < 0.05 were considered statistically significant for both genes and metabolites. The KEGG enrichment results were visualized using the KEGG Automatic Annotation Server (KAAS) [29], and key metabolic pathways were plotted based on the KEGG database.

### 2.5. WGCNA

To elucidate gene expression profiles associated with the early stage of germination in cotton seeds under salt stress, the Weighted Gene Co-expression Network Analysis (WGCNA) package was used to perform a co-expression analysis of the DEG expression profile with the dynamic branch cutting method [32]. The weight coefficient *β* should satisfy a correlation coefficient close to 0.8. In this study, *β* = 9 was selected as the weight coefficient. The network was constructed using blockwise modules to obtain the gene co-expression module (minimum module size = 30; merge cut height = 0.25). The correlation coefficients and *p*-values between the module’s characteristic vector module eigengene (ME) and the salt treatment at different time points were calculated. A similarity matrix between all gene pairs was built using bi-weight mid-correlation based on fragments per kilobase per million reads (FPKMs).

## 3. Results

### 3.1. Phenotypic Characterization of Cotton Seedlings Under Salt Stress

**Figure 1:**
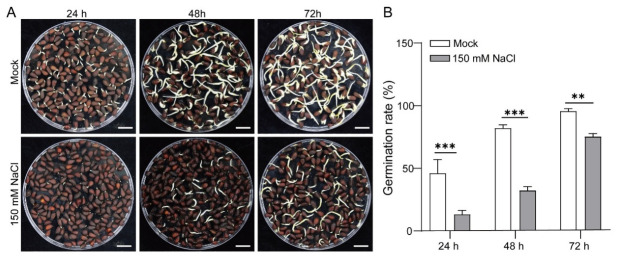
Effects of salt stress on cotton seed germination. (**A**) The germination phenotype of cotton seeds (ST022-1056m5, salt-tolerant cultivar) under conditions of water treatment (Mock) and salt (150 mM NaCl) treatment. Scale bars = 2 cm. (**B**) Temporal changes in seed germination under mock (water) and salt stress (150 mM NaCl) conditions at 24 h, 48 h, and 72 h. Asterisks indicate statistically significant differences, as determined by Student’s *t*-test (** *p* < 0.01, *** *p* < 0.001), and data are presented as the means of at least three biological replicates (±SD).

Seeds are essential for the survival and dispersal of both angiosperms and gymnosperms [21]. Salt-tolerant cotton (*G. hirsutum* cv. ST022-1056m5) seeds were treated with 150 mM NaCl. Seed germination under water control and salt stress was monitored at 24, 48, and 72 h post-treatment (Figure 1A). The results showed that, at 24 h, the germination rate was 48% in the water-treated control group, compared to only 20% in the NaCl-treated group. At 48 h, the germination rate increased to 76% in the control group, while it reached 42% under salt stress. At 72 h, the control group achieved a germination rate of 98%, whereas the NaCl-treated group showed a germination rate of 72% (Figure 1B). In summary, salt stress significantly inhibited cotton seed germination.

### 3.2. Transcriptomic Analysis of Cotton Seed Germination Under Salt Stress

**Figure 2:**
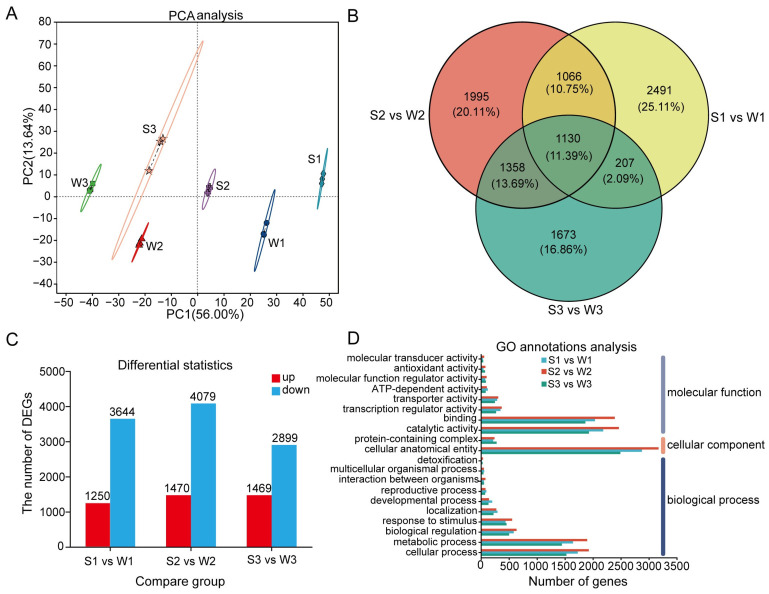
Identification and functional analysis of differentially expressed genes in the early stage of cotton seed germination under salt stress. (**A**) Principal component analysis (PCA) was performed on the transcriptomic data from the following treatment groups: W1 (water treatment for 24 h), S1 (salt treatment for 24 h), W2 (water treatment for 48 h), S2 (salt treatment for 48 h), W3 (water treatment for 72 h), and S3 (salt treatment for 72 h). (**B**) A Venn diagram illustrating the differentially expressed genes (DEGs) across comparison groups: W1, S1, W2, S2, W3, and S3. Each circle corresponds to one distinct comparison. (**C**) The number of DEGs identified in the pairwise comparisons (W1 vs. S1, W2 vs. S2, and W3 vs. S3) is displayed. (**D**) Gene ontology (GO) enrichment analysis of the DEGs from each comparison (W1 vs. S1, W2 vs. S2, and W3 vs. S3). Salt treatment was carried out using 150 mM NaCl.

To identify the key genes involved in cotton (*G. hirsutum* cv. ST022-1056m5) seed germination under salt stress, we performed transcriptome sequencing on seeds treated with 150 mM NaCl. Seeds under water treatment conditions served as the control (mock). Samples were collected at three time points: 24 h, 48 h, and 72 h. Differentially expressed genes were compared across the following treatment groups: W1 (water treatment for 24 h), S1 (salt treatment for 24 h), W2 (water treatment for 48 h), S2 (salt treatment for 48 h), W3 (water treatment for 72 h), and S3 (salt treatment for 72 h). The functional annotation and enrichment analyses were integrated. The principal component analysis (PCA) of gene expression profiles, i.e., PC1 (56.00%) and PC2 (13.64%), collectively explained ~69.64% of the total variance (Figure 2A). Samples from each pairwise comparison (S1 vs. W1, S2 vs. W2, S3 vs. W3) clustered distinctly, indicating robust group-specific transcriptional signatures. A Venn diagram quantified the overlap of differentially expressed genes (DEGs) across the three comparisons: 2491 (25.11%), 1995 (20.11%), and 1673 (16.86%) DEGs were unique to S1 vs. W1, S2 vs. W2, and S3 vs. W3, respectively, 1066 (10.75%) DEGs were shared between S1 vs. W1 and S2 vs. W2, 207 (2.09%) between S1 vs. W1 and S3 vs. W3, 1130 (11.39%) between S2 vs. W2 and S3 vs. W3, and 1358 (13.69%) DEGs were common to all three comparisons (Figure 2B). Then, we analyzed the number of upregulated and downregulated DEGs: S1 vs. W1 exhibited 1250 upregulated and 3644 downregulated genes; S2 vs. W2 had 1470 upregulated and 4079 downregulated genes; and S3 vs. W3 contained 1469 upregulated and 2899 downregulated genes; this analysis revealed a consistent trend of more downregulated transcripts across comparisons (Figure 2C). Gene Ontology (GO) enrichment analysis was performed on differentially expressed genes (DEGs), with results categorized into three functional domains: molecular function, cellular component, and biological process. For biological process, “cellular process” and “metabolic process” were the most highly represented terms across all comparisons; for cellular component, “cellular anatomical entity” and “protein-containing complex” were prominent; and for molecular function, “catalytic activity” and “transporter activity” were enriched (Figure 2D, Tables S1 and S8). These results highlight the functional pathways perturbed in the experimental groups relative to their respective controls.

### 3.3. Transcriptional Profiling and Functional Enrichment Uncover Dynamic Gene Expression Reprogramming Across Salt Stress Treatment Stages

**Figure 3:**
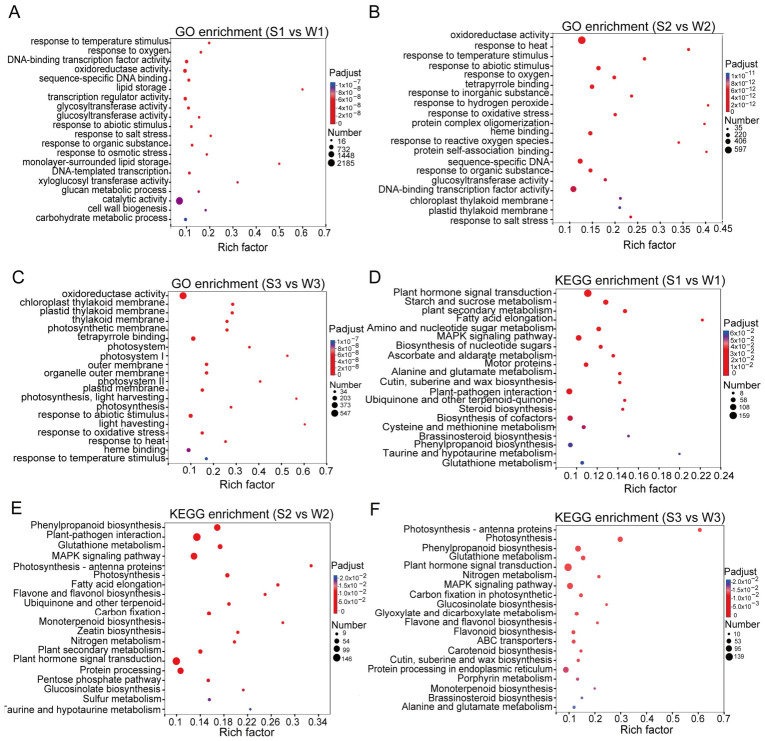
Comprehensive analysis of differentially regulated genes’ enrichment across experimental groups. (**A**–**C**) Gene Ontology (GO) enrichment analysis of the DEGs from the same group comparisons. (**D**–**F**) Kyoto Encyclopedia of Genes and Genomes (KEGG) pathway enrichment for the three comparative groups: W1 vs. S1, W2 vs. S2, and W3 vs. S3. GO enrichment and KEGG enrichment analyses were performed on the transcripts in the gene set using an R script. A significance threshold of an adjusted *p*-value (*p*-adjust) < 0.05 was applied to define statistically significant enrichment.

To further annotate the biological functions of DEGs, we performed GO enrichment analysis (Figure 3A–C, Tables S2 and S8). The plots display GO terms (x-axis: Rich factor, representing the ratio of DEGs to total genes annotated to the term; color: *P*-adjust; size: the number of DEGs in the term). In all three comparisons, DEGs were predominantly enriched in terms related to metabolic processes, cellular component organization, and response to stimuli, suggesting that the treatments primarily modulated fundamental biological activities. For example, in S1 vs. W1 (Figure 3A), top enriched terms included “oxidation–reduction process” and “membrane part”; in S2 vs. W2 (Figure 3B) and S3 vs. W3 (Figure 3C), terms like “protein phosphorylation” and “signal transduction” were prominently represented, implying enhanced regulatory signaling in later treatment stages. We also mapped DEGs to Kyoto Encyclopedia of Genes and Genomes (KEGG) pathways (Figure 3D–F, Table S3) to identify key metabolic and signaling pathways affected by the salt treatments. In the S1 vs. W1 group (Figure 3D), DEGs were enriched in “phenylpropanoid biosynthesis” and “plant hormone signal transduction”, highlighting early changes in secondary metabolism and hormonal regulation. For S2 vs. W2 (Figure 3E) and S3 vs. W3 (Figure 3F), pathways such as the “MAPK signaling pathway” and “carbon metabolism” were significantly enriched, indicating that prolonged treatment amplified the modulation of energy metabolism and stress-responsive signaling cascades.

### 3.4. Differential Accumulation and Regulatory Patterns of Stress-Responsive Metabolites

**Figure 4:**
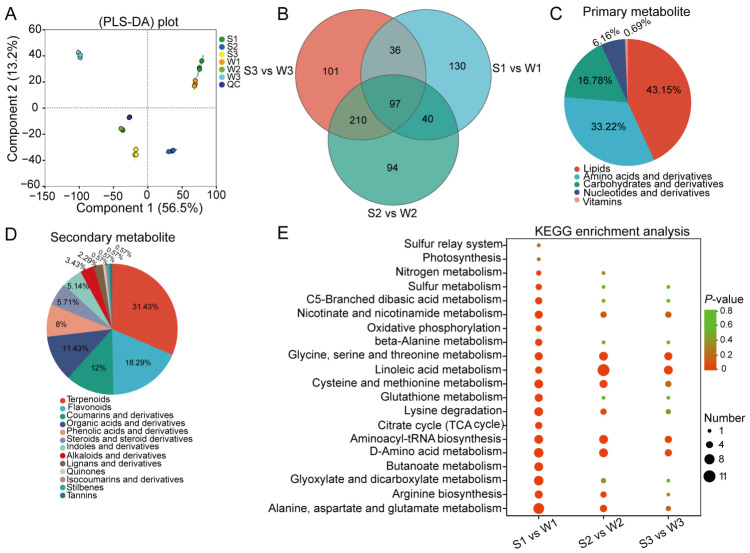
Metabolomic profiling of cotton seeds during early germination under salt stress. (**A**) Partial Least Squares Discriminant Analysis (PLS-DA) of metabolite profiles between control and salt-treated groups. The PLS-DA score plot shows the first two components, which explain 56.5% (Component 1) and 13.2% (Component 2) of the total variance. The distinct clustering of sample points demonstrates significant separation between the control and the six experimental groups. (**B**) A Venn diagram illustrating the overlap of differentially accumulated metabolites (DAMs) identified in the pairwise comparisons: W1 vs. S1, W2 vs. S2, and W3 vs. S3. (**C**) Distribution of primary differential metabolites across the comparison groups, presented as a pie chart. (**D**) Proportion of secondary metabolites in each comparison group, shown in a pie chart. (**E**) KEGG functional annotation and enrichment analysis of the specific metabolites in different groups: W1 vs. S1, W2 vs. S2, and W3 vs. S3. A bubble plot showing enrichment results: circle size indicates the number of DAMs, and color corresponds to the statistical significance (*p*-value).

To dissect the metabolic alterations in cotton seeds during early germination under salt stress, we first characterized metabolite profiles via Partial Least Squares Discriminant Analysis (PLS-DA). The PLS-DA score plot (Figure 4A) showed a clear separation between the control and salt-treated groups, with component 1 (56.5%) and component 2 (13.2%) collectively explaining ~70% of the total variance, thus indicating distinct metabolic reprogramming induced by salt stress. We then identified differentially accumulated metabolites (DAMs) across pairwise comparisons (W1 vs. S1, W2 vs. S2, W3 vs. S3), and their overlap was visualized via a Venn diagram (Figure 4B): 210 DAMs were shared across all three groups, while 130, 94, and 101 DAMs were unique to W1 vs. S1, W2 vs. S2, and W3 vs. S3, respectively. This pattern reflects both conserved and stage-specific metabolic responses to salt stress. The classification of DAMs revealed divergent distributions of primary and secondary metabolites: primary metabolites (Figure 4C) were dominated by “lipids” (43.15%) and “organic acids and derivatives” (33.22%), whereas secondary metabolites (Figure 4D) were enriched in “terpenoids” (31.43%) and “phenolic acids and derivatives” (18.28%), which suggested that salt stress prioritizes adjustments to energy metabolism (primary metabolites) and stress-related defense compounds (secondary metabolites). Finally, KEGG enrichment analysis (Figure 4E) linked DAMs to key pathways: in W1 vs. S1, pathways like “alanine, aspartate and glutamate metabolism” and “citrate cycle (TCA cycle)” were strongly enriched, highlighting early perturbations in central carbon and amino acid metabolism. For W2 vs. S2 and W3 vs. S3, pathways including “glutathione metabolism” and “oxidative phosphorylation” became prominent, indicating a shift toward redox homeostasis and energy production adaptation as germination progressed under salt stress. Together, these results delineate dynamic, stage-dependent metabolic rewiring that supports cotton seed germination in saline environments.

### 3.5. Temporal Dynamics of Differential Metabolites Shape Metabolic Acclimation During Cotton Seed Germination Under Salinity

**Figure 5:**
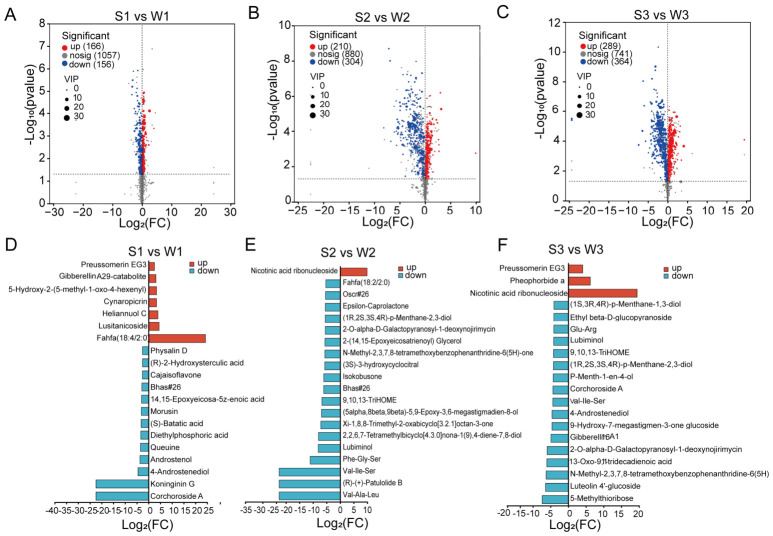
Analysis of differential metabolites during three stages of cotton seed germination under salt stress. (**A**–**C**) Volcano plots depicting the number of differentially accumulated metabolites (DAMs) identified in pairwise comparisons (W1 vs. S1, W2 vs. S2, and W3 vs. S3). Red and blue dots represent up- and downregulated DAMs, respectively. The x-axis shows the log_2_ fold change (log_2_FC), and the y-axis shows the −log_10_ (*p*-value). Significantly differential metabolites were defined as those with a Variable Importance in the Projection (VIP) score from the OPLS-DA model ≥ 1 and a *p*-value < 0.05. (**D**–**F**) Top 20 DAMs ranked by absolute log_2_ fold change (log_2_FC) in each comparison (W1 vs. S1, W2 vs. S2, and W3 vs. S3). Upregulated and downregulated differential metabolites are shown in red and blue, respectively. All compound names are available in the Chemical Entities of Biological Interest (ChEBI) database (https://www.ebi.ac.uk/chebi/, accessed on 22 May 2026).

To characterize stage-specific metabolite dynamics during salt-stressed cotton seed germination, we identified differentially accumulated metabolites (DAMs) across three pairwise comparisons (W1 vs. S1, W2 vs. S2, W3 vs. S3) using the criteria of VIP ≥ 1 (from OPLS-DA) and *p* < 0.05. Volcano plots (Figure 5A–C) visualized these DAMs: in W1 vs. S1 (Figure 5A), 166 metabolites were upregulated and 156 were downregulated; in W2 vs. S2 (Figure 5B), 210 metabolites were upregulated and 304 were downregulated; and in W3 vs. S3 (Figure 5C), 289 metabolites were upregulated and 364 were downregulated. The distribution of dots (red: upregulated; blue: downregulated) highlights a strong bias toward downregulation in the early stages (W1 vs. S1, W2 vs. S2), which shifts toward more balanced regulation by the late stage (W3 vs. S3).

We further prioritized the top 20 DAMs using absolute log_2_ fold change (log_2_FC) for each comparison (Figure 5D–F). In W1 vs. S1 (Figure 5D, Table S7), Preussomerin EG3 (a member of tetralins) and Gibberellin A29-catabolite (a C19-gibberellin) were among the most upregulated metabolites, while R-2-hydroxystearic acid (an aliphatic alcohol) and Bhas#26 (an (1)-hydroxy fatty acid ascaroside) were strongly downregulated, suggesting that early salt stress disrupts phytohormone and lipid metabolism. For W2 vs. S2 (Figure 5E), nicotinic ribonucleoside acid was upregulated, whereas 9,10,13-Trihome (a long-chain fatty acid) and Val-Ala-Leu were the most downregulated, indicating fatty acid derivatives and amino acid metabolism. In the W3 vs. S3 group (Figure 5F), Preussomerin EG3 and nicotinic acid riboside (an orally active NAD^+^ precursor) were highly upregulated, while (1S,3R,4R)-*p*-menthane-1,3-diol (a *p*-menthane monoterpenoid) was the most downregulated, reflecting late-stage adjustments to secondary metabolite and nucleotide metabolism.

### 3.6. Integrated Analysis Reveals Modular Metabolic Reprogramming in Germinating Cotton Seeds

**Figure 6:**
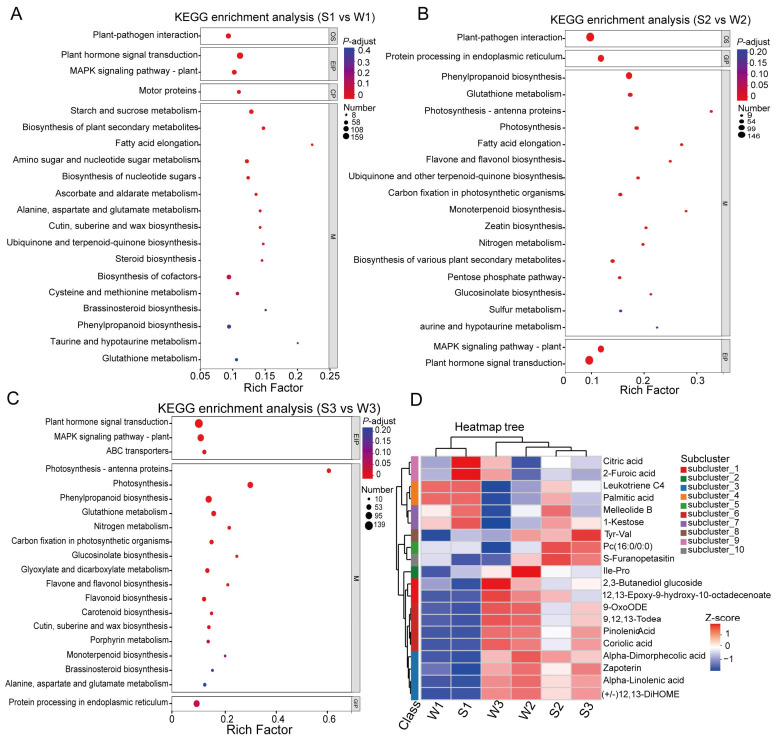
Classification of metabolites and identification of differentially accumulated metabolites (DAMs). (**A**–**C**) Kyoto Encyclopedia of Genes and Genomes (KEGG) pathway enrichment and differential abundance score diagram of DAMs in W1 vs. S1, W2 vs. S2, and W3 vs. S3. The bubble size represents the number of DAMs, and the color corresponds to the *p*-value. OS: oxidative stress; EIP: environmental information processing; CP: cellular processes; GIP: genetic information processing; M: module. (**D**) Genetic information processing. (**D**) Hierarchical clustering and heatmap analysis of the top 20 DAMs. Hierarchical clustering with a distance metric and the complete linkage method, divided into 10 subclusters. Original sample grouping, including the subgroups W1, W2, W3, S1, S2, and S3, and quality control (QC) samples, with group-wise calculation set to “mean value”. Each column in the figure represents a sample, each row represents a metabolite, and the color scale represents the relative abundance of metabolites within each group, with red and blue denoting high and low expression levels, respectively.

To delineate the functional pathways and clustering patterns of DAMs during salt-stressed cotton seed germination, we performed KEGG pathway enrichment analysis for the three pairwise comparisons (W1 vs. S1, W2 vs. S2, W3 vs. S3) (Figure 6A–C and Figure S1, Table S4). In each bubble plot, bubble size corresponds to the number of DAMs mapped to a pathway, while color intensity reflects the *p*-adjust value (darker hues indicate greater significance). For W1 vs. S1 (Figure 6A), DAMs were most strongly enriched in plant hormone signal transduction, indicating the early activation of stress-responsive signaling. In the W2 vs. S2 group (Figure 6B), pathways including “phenylpropanoid biosynthesis” and “flavone and flavonol biosynthesis” became prominent, consistent with enhanced secondary metabolite production for stress tolerance. In the W3 vs. S3 group (Figure 6C), enrichment shifted toward “MAPK signaling pathway-plant” and “ABC transporters”, suggesting the late-stage modulation of signal transduction and metabolite transport to sustain germination.

To further resolve metabolite coregulation, we constructed a heatmap tree to cluster DAMs across all control and salt-treated groups (W1, S1, W2, S2, W3, S3) based on z-score-normalized abundance. The heatmap revealed distinct subclusters with coordinated abundance patterns: for example, metabolites in subcluster 1 (citric acid, 2-furoic acid) exhibited elevated levels in salt-treated groups (S1–S3), while those in subcluster 10 (12,13-epoxy-9-hydroxyl-10-octadecenoate) showed reduced accumulation under salt stress (Figure 6D). This clustering highlights conserved metabolic modules that respond to salt stress, providing insights into the coordinated metabolic networks supporting germination in saline environments. Together, these analyses link DAMs to functionally relevant pathways and coregulated modules, illustrating a dynamic metabolic reprogramming strategy—from early signaling activation to late-stage transport and secondary metabolism enhancement—that enables cotton seeds to adapt to salt stress during germination.

### 3.7. WGCNA Reveals Stage-Specific Co-Expression Modules and Hub Genes Associated with Cotton Seed Germination

**Figure 7:**
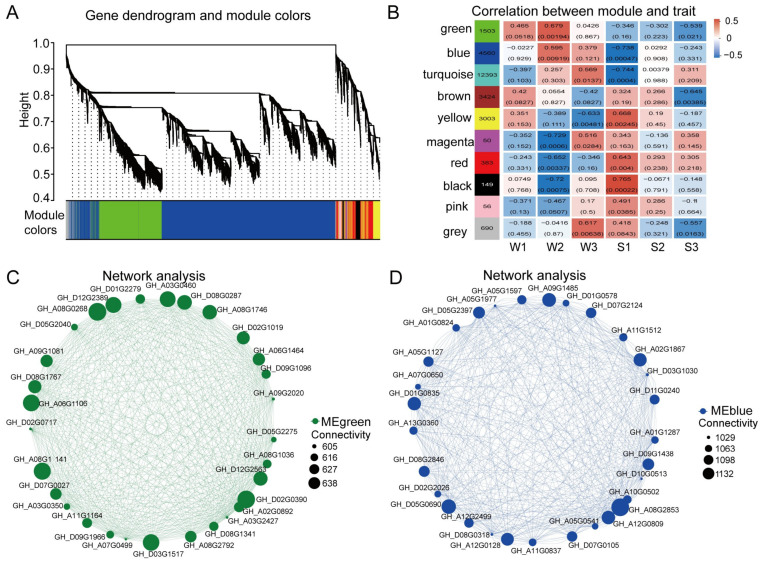
Weighted Gene Co-expression Network Analysis (WGCNA) of differentially expressed genes (DEGs) during cotton seed germination under salt stress. (**A**) Co-expression networks constructed from transcriptome-based DEGs across three germination stages. (**B**) A heatmap depicting module–trait correlations: rows represent module eigengenes (MEs), and columns represent phenotypes. Each cell shows the correlation coefficient and the corresponding *p*-value. Red indicates a positive correlation; blue indicates a negative correlation. Co-expression networks are presented for the green (**C**) and blue (**D**) modules. Nodes represent genes (denoted by their respective gene IDs), edges represent co-expression associations, and node size corresponds to module membership (intramodular connectivity; legend inset).

To dissect the coordinated transcriptional networks underlying salt-stressed cotton seed germination, we performed Weighted Gene Co-expression Network Analysis (WGCNA) on DEGs across three stages. First, a gene dendrogram (Figure 7A) clustered DEGs into distinct co-expression modules, reflecting groups of genes with synchronized expression patterns. We then linked these modules to germination stage traits (W1/W2/W3, S1/S2/S3) via module–trait correlation heatmaps (Figure 7B). Each cell denotes the correlation coefficient between a module eigengene (ME) and a trait: for example, the “green” module showed a strong positive correlation with S1 (correlation = 0.82, *p* = 0.001), while the “blue” module correlated highly with W1 (correlation = 0.90, *p* < 0.001). These associations identify modules functionally linked to salt stress responses at specific germination stages.

To further resolve hub genes within key modules, we constructed network diagrams for the “green” (Figure 7C) and “blue” (Figure 7D) modules: nodes represent genes, edges represent co-expression connections, and node size reflects connectivity. For the “green” module (S1-associated), genes like *GH_A02G0892* emerged as potential regulators of early salt stress responses; *GH_A02G0892* is a zinc finger protein ZAT10-like, which was reported as a salt tolerance zinc finger protein in Arabidopsis (Figure S3, Table S5). For the “blue” module (W1-associated), *GH_A082853* may mediate basal germination processes in non-stressed conditions. *GH_A08G2853* was reported as ubiquitin-specific protease 23 protein in Arabidopsis. These two hub genes were highly induced by salt stress under 150 mM NaCl treatment (Figure S3, Table S5). Collectively, WGCNA reveals stage-specific co-expression modules and hub genes, providing a framework to understand coordinated transcriptional regulation during salt-adapted cotton seed germination.

### 3.8. Integrated Multi-Omics Profiling Reveals Condition-Specific Molecular Responses

**Figure 8:**
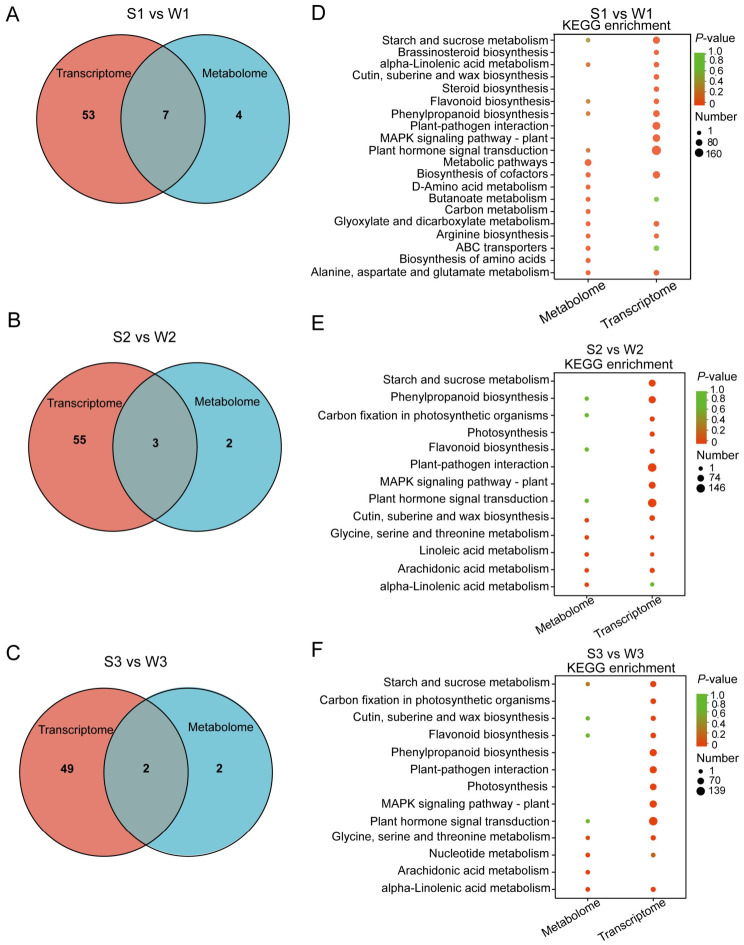
Integrated multi-omics analysis of transcriptomic and metabolomic responses across three experimental conditions (S1, S2, S3). (**A**–**C**) Venn diagrams showing the number of unique and shared significantly altered features between transcriptomics and metabolomics datasets in (**A**) S1, (**B**) S2, and (**C**) S3. Overlapping regions correspond to features that are concordantly altered in both transcriptomic and metabolomic layers. (**D**–**F**) Scatter plots displaying KEGG pathway enrichment results for the significantly altered genes and metabolites in (**D**) S1, (**E**) S2, and (**F**) S3. The x-axis indicates the omics layer (transcriptome or metabolome) contributing to pathway enrichment, the y-axis lists the enriched KEGG pathways, the color gradient represents the statistical significance (*p*-value), and the dot size reflects the number of genes or metabolites mapped to each pathway.

To dissect the molecular underpinnings of phenotypic divergence across the three experimental conditions (S1, S2, S3), we performed an integrated analysis of transcriptomic and metabolomic datasets. Venn diagram analysis was performed to quantify the overlap between significant DEGs and DAMs (Figure 8A–C, Table S4). Condition S1 exhibited the highest degree of multi-omics integration, with seven biological pathways showing concordant changes at both transcript and metabolite levels, compared to only three and two overlapping pathways in S2 and S3, respectively. This indicates that S1 engages a more tightly coupled transcriptional and metabolic regulatory network, whereas S2 and S3 rely on more distinct omics-level responses.

KEGG pathway enrichment analysis further resolved the functional pathways driving these condition-specific responses (Figure 8D–F and Figure S2). Under salt stress for 24 h (S1), starch and sucrose metabolism, phenylpropanoid biosynthesis, and the MAPK signaling pathway were significantly enriched in both transcriptomic and metabolomic profiles. The coordinated upregulation of genes and metabolites in starch and sucrose metabolism suggests increased carbon flux through primary metabolism, while phenylpropanoid enrichment indicates the activation of stress-related secondary metabolism. Concurrent MAPK pathway enrichment further supports regulation by stress-responsive signaling (Figure 8D). In S2 (salt stress for 48 h), enrichment shifted toward energy acquisition pathways, including photosynthesis and photosynthetic carbon fixation, reflecting prioritized light conversion consistent with enhanced photosynthetic activity. Enrichment of flavonoid biosynthesis also implied the activation of photoprotective mechanisms (Figure 8E). S3 (salt stress for 72 h) showed enrichment in cutin, suberin, and wax biosynthesis, along with porphyrin and chlorophyll metabolism. The upregulation of cuticular lipid pathways indicates active cuticle remodeling, which is likely to limit water loss or pathogen entry. Changes in porphyrin and chlorophyll metabolism suggest modifications of the photosynthetic apparatus, possibly in response to altered light or oxidative stress (Figure 8F). Taken together, these results indicate distinct transcriptional and metabolic adaptations under each condition; among all samples, S1 demonstrated the most robust and coordinated shifts in both transcriptomic and metabolomic profiles.

## 4. Discussion

Salt stress is a major abiotic constraint on cotton production, with the early seed germination stage (0–72 h) representing the most vulnerable window for seedling establishment [33,34]. During this phase, seeds transition from dormancy to active metabolism, and salt interference, water uptake, ion homeostasis, and energy metabolism critically determine subsequent growth vigor [15,16,35]. Understanding the molecular and physiological response mechanisms during this “golden window” is thus pivotal for breeding salt-tolerant cotton varieties and ensuring production security in saline–alkali soils [17,36]. Current research predominantly focuses on later germination stages or seedling growth, leaving a critical gap in understanding high-spatiotemporal-resolution dynamic responses during the initial 0–72 h, when seed coat rupture and metabolic reactivation and morphogenesis initiation occur [19,21,26,37,38]. To address this gap, we integrated multi-omics approaches such as transcriptomics and metabolomics with systematic sampling (0–72 h) across cotton varieties with varying salt tolerance under gradient NaCl stress.

Integrated pathway and clustering analyses revealed a distinct, three-stage metabolic reprogramming strategy that underpins salt tolerance during cotton seed germination. In early imbibition (W1 vs. S1), the significant enrichment of DAMs in plant hormone signal transduction pathways indicates that the primary response to salinity is the rapid modulation of hormonal networks to counteract stress-induced dormancy. As germination progresses (W2 vs. S2), the metabolic focus shifts toward biochemical fortification, evidenced by the upregulation of phenylpropanoid and flavonoid biosynthesis, which likely enhances antioxidant defenses and reinforces cell wall integrity. In the later stages (W3 vs. S3), enrichment of the MAPK signaling pathway and ABC transporters suggests a transition to maintaining cellular homeostasis through refined signal transduction and active metabolite transport [21,38,39]. Complementing these findings, heatmap clustering identified conserved metabolic modules, such as the sustained elevation of citric acid and 2-furoic acid, that function coordinately to support germination under saline conditions. Notably, the functional categories of these salt-responsive DAMs showed significant consistency with previous metabolic studies for cotton seed germination under salt stress [19,37].

Key findings reveal the dynamic reprogramming of gene expression (DEGs) and metabolites (DAMs) during the early stage of germination. DEGs and DAMs were significantly enriched in reactive oxygen species (ROS) metabolism, plant hormone signaling, and amino acid biosynthesis pathways [40,41,42]. WGCNA identified hub genes and modules tightly correlated with salt tolerance traits, constructing a molecular regulatory network underlying salt-tolerant germination. These results uncover core metabolic pathways (e.g., ROS scavenging, ion compartmentalization) and potential regulatory hubs (e.g., transcription factors linked to hormone balance and cell wall plasticity) critical for cotton’s early salt adaptation, which are consistent with previous studies [42,43,44,45,46,47].

Our study highlights the power of integrated multi-omics in resolving the “black box” of early salt stress responses, advancing beyond static or low-resolution analyses [26,37]. By linking transcriptional and metabolic dynamics, we provide a theoretical framework for targeted functional validation (e.g., CRISPR-Cas9 editing of hub genes), applied molecular marker-assisted selection, genetic engineering for salt tolerance, and optimized seed treatments to enhance emergence vigor in saline soils [21,38].

Study limitations include reliance on controlled laboratory conditions and the need for broader genotype validation. Future work should prioritize the following: high-resolution temporal studies (0–72 h) tracking physiological–biochemical–molecular–metabolic networks; integrating multi-omics with live-cell imaging for spatiotemporally precise response mapping; mechanistic validation of key genes and pathways via genetics and molecular biology [7,39]; and translating findings into scalable breeding and cultivation strategies [48]. Without a comparative framework contrasting the tested salt-tolerant cultivar with a genetically related one, it is difficult to separate salt-specific tolerance from general stress responses shared by all plants under salinity. Despite this limitation, our findings contribute to the growing body of knowledge on salt stress responses in cotton seeds by providing a detailed baseline of physiological and molecular changes. In summary, this study establishes a foundation for understanding salt-tolerant cotton’s early germination, offering genetic resources and theoretical insights to improve saline–alkali land utilization and sustainably secure cotton production under climate change.

## 5. Conclusions

In conclusion, this study systematically elucidated the stage-dependent salt tolerance mechanisms in the cotton cultivar ST022-1056m5 during seed germination under NaCl stress via integrated multi-omics analyses. We revealed a dynamic adaptation strategy: the early activation of stress signaling and primary metabolic adjustment, mid-stage enhancement of energy production and secondary metabolism, and late-stage optimization of structural protection and photosynthesis. Transcriptomic profiling identified core DEGs enriched in metabolic processes, MAPK signaling, and hormone regulation, while metabolomic data uncovered stage-specific DAMs (primary/secondary metabolites) driving redox homeostasis and energy adaptation. WGCNA further highlighted stage-specific co-expression modules and hub genes (e.g., *GH_A02G0892*, *GH_A08G2853*) as key candidates. Tight transcriptional–metabolic coupling in the early stage of germination, transitioning to specialized responses later, underscored phase-specific regulatory logic. These identified DEGs, DAMs, and hub genes provide valuable molecular resources for breeding salt-tolerant cotton. Future research will focus on the functional validation of key regulators to enhance germination resilience in saline environments.

## Data Availability

The transcriptomic data in the fastq format used in this study can be found in the database (Accession ID: PRINA1335884); the compound data obtained from metabolomic analysis can be found in Supplementary Table S4.

## Supplementary Materials

The following supporting information can be downloaded at: https://www.mdpi.com/article/10.3390/genes17070761/s1, Figure S1: The score plot of principal component analysis (PCA) for three distinct sample comparison sets. Figure S2: Differential metabolism across groups and KEGG pathway enrichment heatmap. Figure S3: qRT-PCR validation of selected hub gene expression level under salt stress. Table S1: Details of the GO analysis. Table S2: Top 20 enriched GO terms’ analysis. Table S3: The top 20 enriched KEGG pathways. Table S4: Characterization of differential metabolite profiles. Table S5: Gene lists of the green and blue co-expression modules identified using WGCNA. Table S6: Number of raw sequencing reads and post-quality control (QC) reads for each sample. Table S7: Details of the top 20 differential metabolites. Table S8: Expression patterns of up- and downregulated DEGs.
