# Supplementary material for: Integrated Transcriptome and Metabolome Analysis Elucidates the Regulatory Networks of Salt Stress Response During Cotton Seed Germination
Source: Genes (Basel). 2026 Jun 30;17(7):761. doi: 10.3390/genes17070761 (PMC13409522; doi:10.3390/genes17070761)
Supplement: Supplementary file 1 [file genes-17-00761-s001.zip › supplementary figures.pdf]

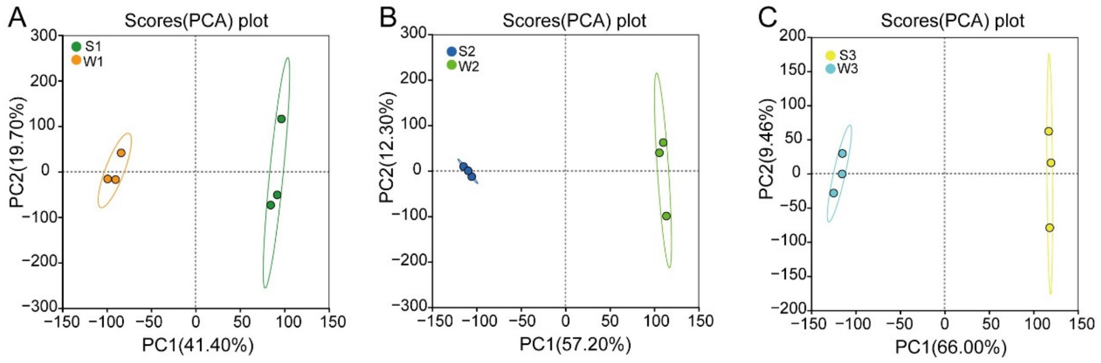

Figure S1. The scores plot from Principal Component Analysis (PCA) for three distinct sample comparison sets. (A) Panel A (S1 vs. W1): This PCA scores plot illustrates the distribution of S1 and W1 samples along the first two principal components (PCs). PC1 explains 41.40% of the total variance, while PC2 accounts for 19.70% of the variance. (B) For the S2 vs. W2 comparison, PC1 contributes 57.20% of the total variance, and PC2 contributes 12.30% of the variance. (C) For the S3 vs. W3 comparison, PC1 explains 66 % of the total variance, and PC2 explains 9.46% of the variance. The score points of S3 and W3 samples also show distinct separation along the PCs, indicating clear differences between the two groups.

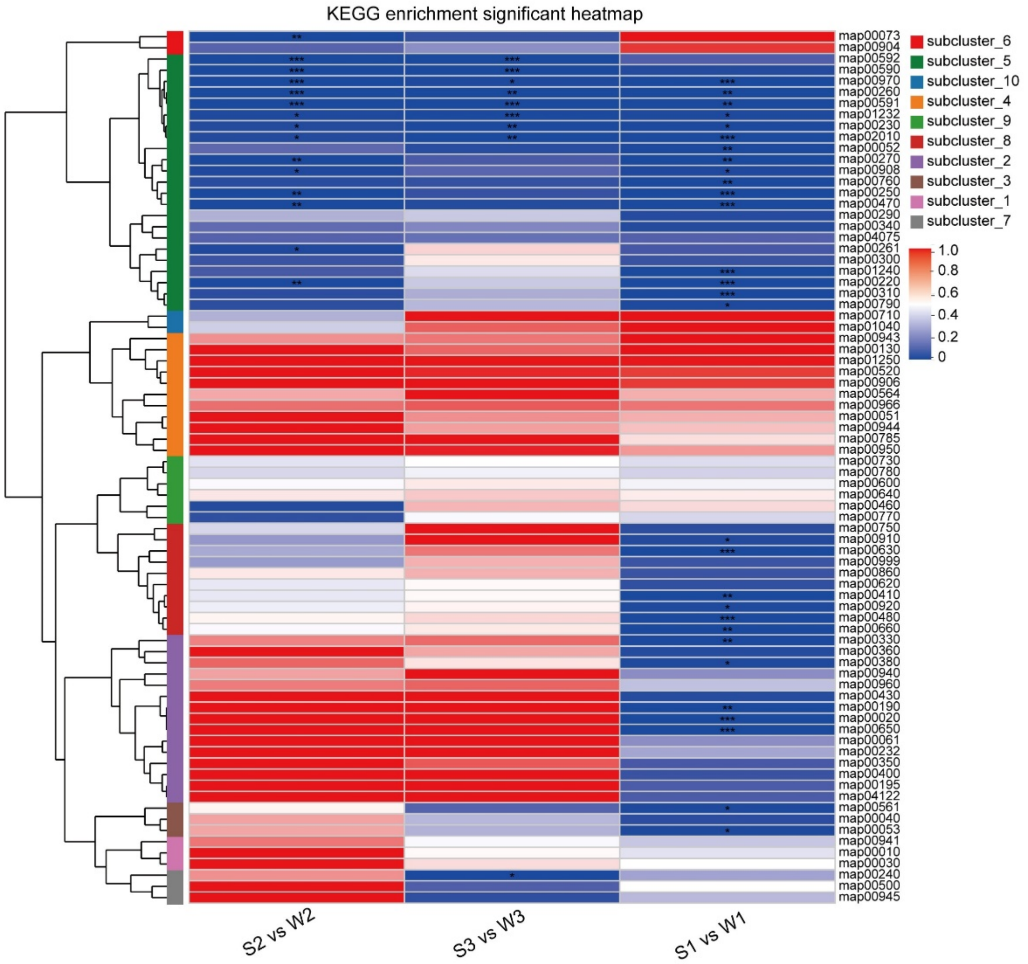

Figure S2. Differential metabolism across groups and KEGG pathway enrichment heatmap. The KEGG enrichment significance heatmap, which illustrates the enrichment significance distribution of KEGG pathways across 10 metabolism subclusters (subcluster_1 to subcluster_10) and three sample comparison groups (S1 vs W1, S2 vs W2, S3 vs W3). Right-side labels: Correspond to KEGG pathways. Bottom columns: Represent the three sample comparison groups (S1 vs W1, S2 vs W2, S3 vs W3). Left hierarchical clustering tree: Reflects the similarity-based clustering of metabolism subclusters, grouped by their KEGG enrichment profiles. The “*” markers indicate that the corresponding enrichment results satisfy the pre-specified statistical significance threshold (*, adjusted $p < 0.05$, **, adjusted $p < 0.01$, ***, adjusted $p < 0.001$).

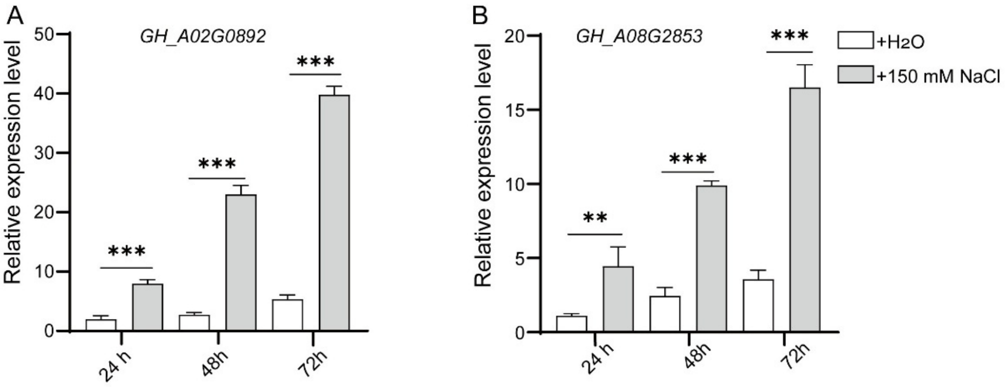

Figure S3. qRT-PCR validation of selected hub genes expression level under salt stress. (A) The expression of *GH_A02G0892* was assessed using forward primer (F): ACTAAGGGTAAGCGTTCTAAGC and reverse primer (R): GGCCTTGTTGCAAACTTTGC, (B) Experiments on *GH_A08G2853* was conducted with forward primer (F): CAGTATCAGTGCCAACGCAT and reverse primer (R): AAACAGTCCTCTCACTGACC. *GhUBQ7* (F: GAAGGCATTCCACCTGACCAAC; R: CTTGACCTTCTTCTTCTTGTGCTTG) was used as a reference gene for normalization purposes. Asterisks indicate statistically significant differences as determined by Student's *t*-test (**$P < 0.01$, ***$P < 0.001$), data are presented as the mean.
